# Machine learning‐based prediction of 1‐year mortality in hypertensive patients undergoing coronary revascularization surgery

**DOI:** 10.1002/clc.23963

**Published:** 2023-01-01

**Authors:** Amir Hossein Behnoush, Amirmohammad Khalaji, Malihe Rezaee, Shahram Momtahen, Soheil Mansourian, Jamshid Bagheri, Farzad Masoudkabir, Kaveh Hosseini

**Affiliations:** ^1^ Tehran Heart Center, Cardiovascular Diseases Research Institute Tehran University of Medical Sciences Tehran Iran; ^2^ Cardiac Primary Prevention Research Center, Cardiovascular Diseases Research Institute Tehran University of Medical Sciences Tehran Iran; ^3^ School of Medicine Tehran University of Medical Sciences Tehran Iran; ^4^ Non‐Communicable Diseases Research Center, Endocrinology and Metabolism Population Sciences Institute Tehran University of Medical Sciences Tehran Iran; ^5^ School of Medicine Shahid Beheshti University of Medical Sciences Tehran Iran; ^6^ Department of Surgery, Tehran Heart Center Tehran University of Medical Sciences Tehran Iran

**Keywords:** coronary artery bypass, hypertension, machine learning, mortality, prediction

## Abstract

**Background:**

Machine learning (ML) has shown promising results in all fields of medicine, including preventive cardiology. Hypertensive patients are at higher risk of mortality after coronary artery bypass graft (CABG) surgery; thus, we aimed to design and evaluate five ML models to predict 1‐year mortality among hypertensive patients who underwent CABG.

**Hyothesis:**

ML algorithms can significantly improve mortality prediction after CABG.

**Methods:**

Tehran Heart Center's CABG data registry was used to extract several baseline and peri‐procedural characteristics and mortality data. The best features were chosen using random forest (RF) feature selection algorithm. Five ML models were developed to predict 1‐year mortality: logistic regression (LR), RF, artificial neural network (ANN), extreme gradient boosting (XGB), and naïve Bayes (NB). The area under the curve (AUC), sensitivity, and specificity were used to evaluate the models.

**Results:**

Among the 8,493 hypertensive patients who underwent CABG (mean age of 68.27 ± 9.27 years), 303 died in the first year. Eleven features were selected as the best predictors, among which total ventilation hours and ejection fraction were the leading ones. LR showed the best prediction ability with an AUC of 0.82, while the least AUC was for the NB model (0.79). Among the subgroups, the highest AUC for LR model was for two age range groups (50–59 and 80–89 years), overweight, diabetic, and smoker subgroups of hypertensive patients.

**Conclusions:**

All ML models had excellent performance in predicting 1‐year mortality among CABG hypertension patients, while LR was the best regarding AUC. These models can help clinicians assess the risk of mortality in specific subgroups at higher risk (such as hypertensive ones).

## 1 INTRODUCTION

Cardiovascular diseases (CVDs) are responsible for approximately 17.9 million deaths annually.^1^ Ischemic heart disease (IHD) is the most prevalent CVD in the general population, as 49.2% of CVD deaths are among IHD patients.^2^ Revascularization methods, including percutaneous coronary intervention (PCI) and coronary artery bypass grafting (CABG), are the primary therapies in IHD.^3^ CABG is one of the most common cardiac surgeries, considered the preferable therapeutic approach in patients with multivessel or left main coronary artery disease (CAD) or in case of left ventricular dysfunction.^4^

With the prevalence of one in every three adults in the United States,^5^ hypertension is a major modifiable risk factor for CAD irrespective of sex and age.^6^ Hypertensive patients tend to have different risk factor patterns from other CABG patients.^7^ Moreover, increased postoperative complications, early mortality, and 2‐year mortality have been reported, compared to nonhypertensive patients.^7^, ^8^ This was reported to be an up to 40% increase in perioperative morbidity in hypertensive patients undergoing CABG.^9^

Besides traditional risk scores, machine learning (ML)‐developed models are getting attention for outcome prediction after cardiac surgeries.^10^ However, there are controversies about the accuracy of ML models compared to risk scores currently being used.^11^ Knowing the greater need for mortality prediction in the hypertensive population, we aimed to use and compare different ML methods to predict 1‐year mortality of hypertensive patients after isolated CABG.

## 2 METHODS

### 2.1 Study design and data collection

We conducted this serial cross‐sectional study based on the Tehran Heart Center CABG registry among hypertensive patients between 2005 and 2015. Hypertension was defined as systolic blood pressure (SBP) ≥ 140 mmHg and/or diastolic blood pressure (DBP) ≥ 90 mmHg following two separate examinations in patients' history and/or taking antihypertensive medications. All the perioperative data of patients were collected and managed by expert nurses in our center. The ethics committee of Tehran Heart Center approved this study (IR.TUMS.THC.1401.023).

### 2.2 Variables' definition

Baseline characteristics including demographic, preoperative, and intraoperative variables were used as potential predictors. Age, gender, weight, height, and body mass index (BMI) were demographics. Serum hemoglobin (Hb), high‐density lipoprotein cholesterol (HDL‐C), low‐density lipoprotein cholesterol (LDL‐C), total cholesterol, triglycerides (TG), and creatinine, in addition to left ventricular ejection fraction (EF) measured by echocardiogram, diabetes, opium consumption, smoking status, prior myocardial infarction (MI), preoperative heart failure (HF), and chronic obstructive pulmonary disease (COPD) were preoperative variables. Finally, hospitalization parameters and intraoperative variables were total ICU hours, total ventilation hours, and cardiopulmonary pump utilization (on‐pump or off‐pump). All these data were obtained from either past medical records or blood sample measurements during hospitalization episodes and before surgery.

### 2.3 Main outcome

The study's main outcome was 1‐year mortality post‐CABG, for which we compared different ML‐based prediction models. This outcome included both in‐hospital and after‐discharge mortality events.

### 2.4 Data cleaning

Exclusion criteria were missing data in addition to out‐of‐range values such as: (1) Hb > 25 g/dl or Hb < 5 g/dl, (2) LDL‐C > 400 mg/dl, (3) TG > 1200 mg/dl or TG < 20 mg/dl, (4) HDL‐C < 5 mg/dl or HDL‐C > 100 mg/dl, and (5) creatinine > 15 mg/dl or creatinine < 0.2 mg/dl were excluded. As we had sufficient data to develop and test models, excluding missing data were possible.

### 2.5 Test/train split, feature selection, and oversampling

In a random assignment process, the total hypertensive population was divided into train and test cohorts (70% and 30%, respectively). The test cohort sample was used to evaluate and validate the ML models.

To select the best predictors for mortality in the total population and each of the subgroups, a feature selection process based on the random forest (RF) model was designed using 10‐fold cross‐validation. This technique investigates the effect of each predictor alone and in combination with other predictive variables. RF feature selection works based on mean decrease accuracy (MDA) and the mean decrease gini (MDG). The former shows how much accuracy is lost if a variable is excluded, while MDG represents the contribution of each variable to the homogeneity of the nodes and leaves in the resulting RF. The higher these scores, the higher the importance of variable.^12^, ^13^, ^14^ Wherever there was a strong clinical and statistical correlation between two variables, the one with better prediction potential and/or clinical relevance was chosen, and the other was omitted.

Our study population was completely imbalanced in terms of mortality, where its rate was only 3.39%. To tackle this common challenge in ML models, we performed the synthetic minority oversampling technique (SMOTE) to balance our data in the training sample. SMOTE works by identifying the minority group's *k*‐nearest neighbors, and it selects a set of neighbors which then generates new data using them.^15^ Ten‐fold cross‐validation with the SMOTE of 25% (for the ratio of the minority to majority group) was used to tune this oversampling strategy and select the best minority to majority class ratio.

As the last step of preparation for the model development, the “standard scaler” (from the scikit‐learn package^16^) was used to scale each variable by removing the mean and scaling to unit variance, which is the requirement for many ML algorithms.

### 2.6 Model development

Predictive ML models used in this study were (1) logistic regression (LR), (2) extreme gradient boosting (XGB), (3) naïve Bayes (NB), (4) RF, and (5) Artificial Neural Network (ANN). The diagram for ANN and the number of layers are shown in Supporting Information: Figure 1. In all models, we used variables obtained by the feature selection method previously described. The "Grid Search” method was used to select the best parameters in each model to increase the accuracy of the model performance.

### 2.7 Model performance evaluation

Performance evaluation was done using the following metrics: A) sensitivity and specificity; B) accuracy of prediction using 10‐fold cross‐validation; C) AUC score by plotting true positive against false positive rate. The threshold is the cut‐off to allocate a probability into a class label and is normally set at 0.5 (50%). Due to the highly imbalanced outcome in our study, this rate of 0.5 was tuned by utilizing 10‐fold cross‐validation in the train data to adjust the sensitivity and specificity of models.

The primary metric for evaluating models was chosen as AUC (with a 95% confidence interval [CI] using several random states) since it is independent of the threshold. To validate the findings, the best model in terms of AUC was implemented to measure the metrics for the most recent 30% of cases in terms of admission time (2013–2015). This method assesses the temporal validity of findings over time.^17^, ^18^

### 2.8 Statistical analysis

**Figure 1:**
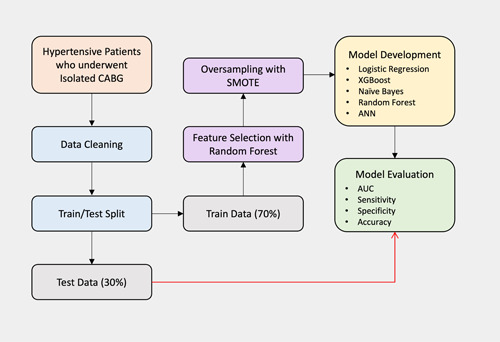
Design of study, including all mentioned steps in ML models. ANN, artificial neural network; AUC, area under the curve; CABG, coronary artery bypass grafting; ML, machine learning; SMOTE, synthetic minority oversampling technique.

Baseline characteristics are reported as mean ± standard deviation (SD) or proportion (percentage). The comparison was made using Pearson *χ* ^2^ test and Fisher's exact test for categorical variables, in addition to an independent sample *t*‐test for continuous variables. A two‐sided *p* value of less than .05 was considered statistically significant. Prediction models were designed and evaluated for 1‐year mortality for the whole hypertensive cohort of patients and subgroups based on gender, age group, BMI, diabetes, and smoking status. All statistical analyses and model development were performed using Python (version 3.10). LR, NB, and RF models were implemented using scikit‐learn (1.0.2) library,^16^ ANN with TensorFlow (version 2),^19^ and XGB using XGBoost (version 1.6.0) Python library. The methodological design of the study including all the mentioned stages performed is illustrated in Figure 1.

## 3 RESULTS

### 3.1 Baseline characteristics

**Figure 2:**
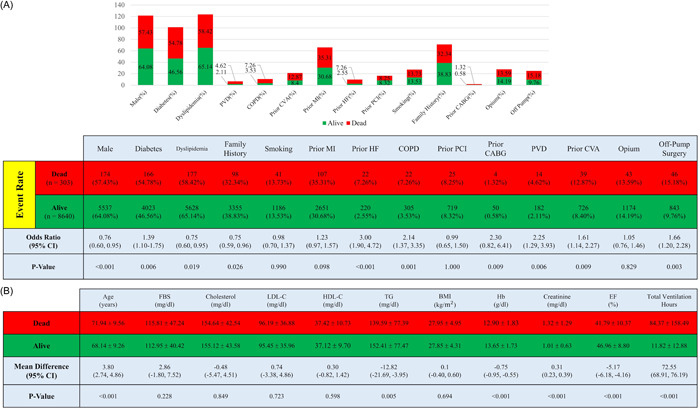
Comparison of baseline and hospitalization characteristics of survivors and nonsurvivors with 1‐year follow‐up; (A) dichotomous variables, (B) continuous variables. BMI, body mass index; CABG, coronary artery bypass grafting; COPD, chronic obstructive pulmonary disease; CVA, cerebrovascular accident; EF, ejection fraction; FBS, fasting blood glucose; Hb, hemoglobin; HDL‐C, high‐density lipoprotein cholesterol; HF, heart failure; LDL‐C, low‐density lipoprotein cholesterol; MI, myocardial infarction; PCI, percutaneous coronary intervention; PVD, peripheral vascular disease; TG, triglyceride.

Totally, 8,493 hypertensive patients with a mean age of 68.27 ± 9.27 years (mean ± SD) were assessed for model development and evaluation. Of the mentioned population, 63.86% were male, 46.84% had diabetes, and 38.61% had a family history of CAD. Details of baseline characteristics of hypertensive patients in the CABG cohort are shown in Supporting Information: Table 1. Among all patients, 303 (3.39%) died during a 1‐year follow‐up. Patients who died were significantly older than survivors (71.94 ± 9.56 vs. 68.41 ± 9.26 years; *p* < .001). Hb and EF were significantly lower in dead patients compared to alive ones. In addition, the prevalence of diabetes was higher in patients who died (54.78% vs. 45.65%; *p* < .001). Figure 2 represents the baseline characteristics of the dead and alive patients in the whole cohort measured before, during, or after the CABG.

### 3.2 Feature selection

**Figure 3:**
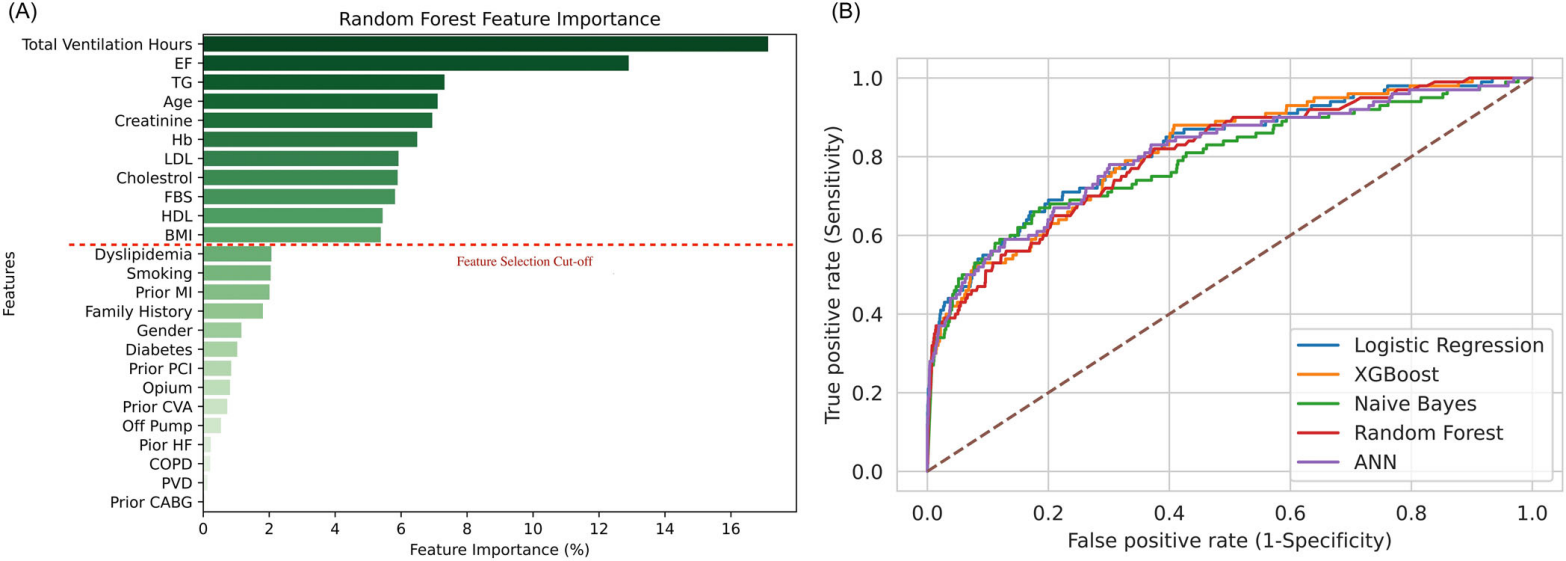
Main findings of the ML algorithm for prediction of 1‐year mortality in patients undergoing CABG; (A) feature importance based on the random forest model, (B) receiver operating characteristic curve for 1‐year mortality prediction in all five ML models. CABG, coronary artery bypass grafting; ML, machine learning.

The RF feature selector was used using a 10‐fold cross‐validation method to select top features given their AUC. Using Pearson correlation *r*, we determined correlations between the features. Total ventilation hours and BMI were used instead of total ICU hours and weight due to statistical correlation and more clinical acceptance. Figure 3A illustrates all sorted feature importance obtained by the RF feature selector and the feature selection cut‐off line. The top variables used in our models were total ventilation hours, EF, TG, age, creatinine, Hb, LDL‐C, total cholesterol, FBS, HDL‐C, and BMI, respectively.

### 3.3 Models evaluation

We designed five ML algorithms for the prediction of 1‐year mortality among hypertensive patients undergoing CABG. Table 1 compares the sensitivity, specificity, and AUC of prediction models. All the models had an acceptable performance with LR outperforming others [AUC (95% CI) = 0.82 (0.78–0.86)]. Considering AUC as the main metric for evaluation, LR was followed by XGB, ANN, RF, and NB. In addition, LR had the highest specificity and accuracy (specificity = 83% and accuracy = 82.37%), while XGB had the best performance in terms of sensitivity (88%). Figure 3B demonstrates the receiver operating characteristic curve (ROC) for all five models. Finally, the LR model showed an AUC of 0.77 (0.73–0.81) for the most recent 30% of the total cohort.

**Table 1:** Evaluation of ML models for the prediction of 1‐year mortality

	Sensitivity (%)	Specificity (%)	Accuracy (%)	AUC [95% confidence Interval]
Logistic regression	66.00	83.00	82.37	**0.819 [0.777–0.863]**
Extreme gradient boosting	88.00	59.23	60.31	0.812 [0.765**–**0.854]
Random forest	76.00	69.34	69.59	0.804 [0.759**–**0.846]
Naïve Bayes	67.00	81.49	80.95	0.791 [0.739**–**0.845]
Artificial neural network	78.00	69.92	70.22	0.806 [0.759**–**0.853]

Abbreviations: AUC: area under the curve; ML, machine learning.

### 3.4 Subgroups

**Figure 4:**
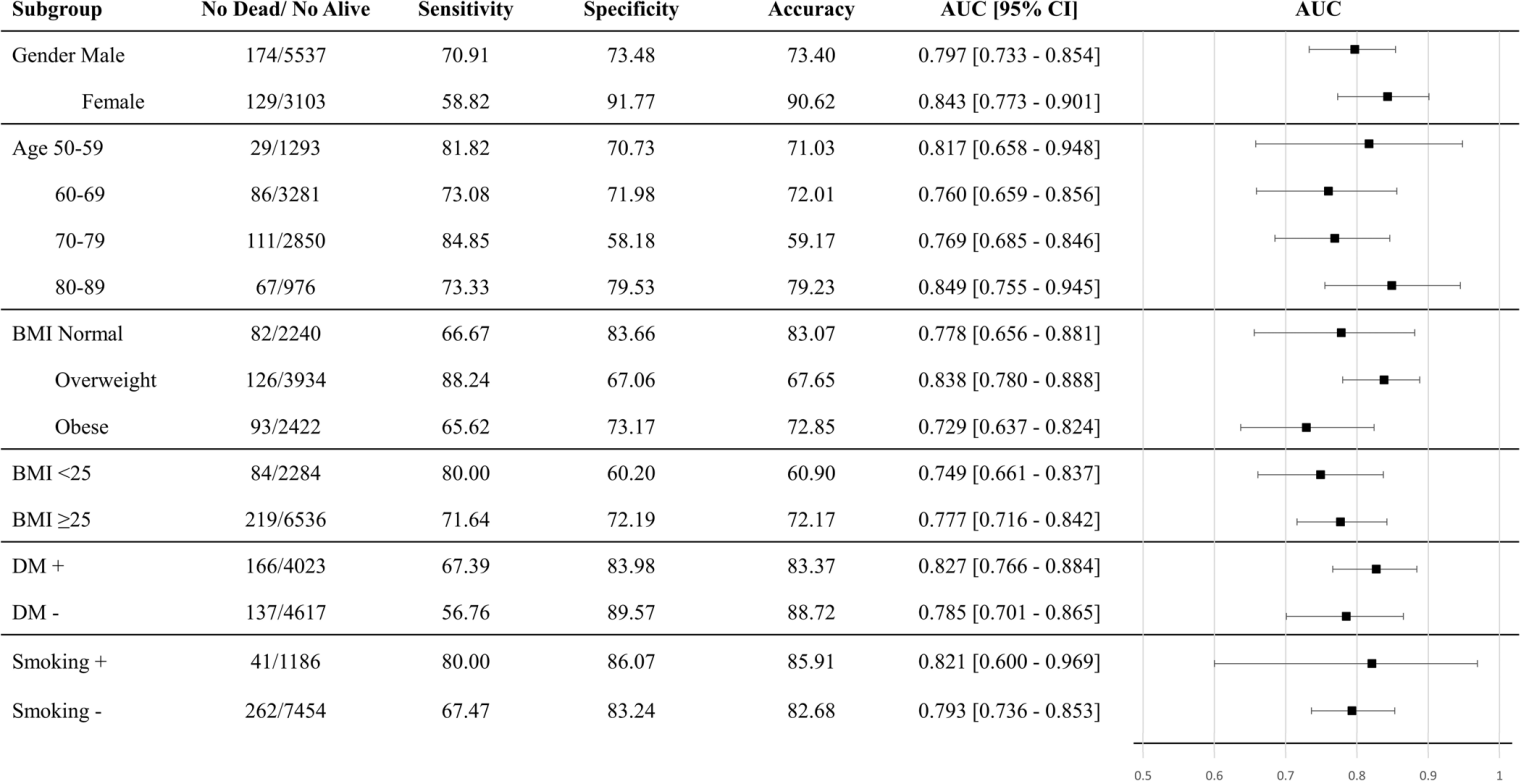
Logistic regression (LR) model evaluation for prediction of mortality in different subgroups of patients

Models ran for each of the subgroups of hypertensive patients. Evaluation metrics for the LR model as the top prediction model in the whole population are illustrated in Figure 4. LR model had the highest prediction ability for females, the age range of 50–59 and 80–89, overweight patients, diabetic cases, and smokers with all having AUC > 0.8 (classified as excellent^20^). The highest sensitivity was for the overweight subgroup of patients, while the highest specificity and accuracy were for the female subgroup. Details of the other four models' evaluations are illustrated in Supporting Information: Figures 2–5. Among all prediction models, the best performance in terms of AUC was for the age subgroup of 50–59 and NB [AUC (95% CI) = 0.863 (0.704–0.988)].

## 4 DISCUSSION

In the present study, we attempted to apply and compare five alternative ML algorithms (LR, XGB, ANN, RF, and NB) concerning the prediction of 1‐year mortality among hypertensive patients undergoing CABG. The findings of this study clearly illustrated the power of ML in improving the prediction of 1‐year mortality, with all five ML models found to be able to predict 1‐year mortality with most of them showing AUC > 0.8 which demonstrates excellent predictive ability, according to AUC interpretation.^20^ LR model generally outperformed the remaining methods and exhibited the greatest discrimination (AUC = 0.82).

Accordingly, accurate mortality risk prediction systems can play an essential role in improving the continuity of care and management after cardiac surgery, leading to an increase in the survival of patients. Several risk scoring tools, such as Society of Thoracic Surgeons (STS), EURO‐Score I and II, have been developed to predict mortality and detect the involved factors. However, several limitations of these scores in some surgeries or patient subgroups have been reported. Therefore, it seems that these scoring tools need to be modified and improved.^21^, ^22^, ^23^, ^24^ The recent advancement in electronic medical records and artificial intelligence resulted in an increasing interest in utilizing ML algorithms for individualized clinical decision‐making and risk prediction.^25^ ML algorithms showed a notable ability to be trained to develop personalized risk prediction scoring systems for outcomes of specific therapeutic approaches such as surgeries by identifying complex patterns in the big data.^26^, ^27^, ^28^ In addition, ML models allow for adjustment of the sensitivity and specificity of each model in different clinical settings in the context of risk predictions at the individual level.^29^ Although some previous studies used the 50% default threshold, which can lead to a plethora of missed cases, we modified it to achieve the optimum sensitivity and specificity on the ROC curve, the same as what was done earlier in other studies.^29^, ^30^

It has been illustrated that ML could improve the forecasting quality of the traditional epidemiologic standard mortality models.^31^, ^32^ These findings extend several studies where they demonstrated the superiority of ML models compared to classical tools for identifying patients at increased risk of mortality after CABG. ML models demonstrated that they could be more accurate in forecasting in‐hospital mortality after cardiac surgeries than EURO‐Score II.^33^ The preoperative ML models also outperformed the conventional STS model concerning the prediction of mortality or major morbidity in patients who underwent isolated CABG, mainly using intraoperative parameters such as cross‐clamp and bypass times as additive predictive factors.^34^ In agreement with these findings, a recent study demonstrated the better performance and validity of eight ML models for predicting 4‐year mortality after cardiac surgery compared to traditional statistical methods. Further analyses showed that adapting boosting (Ada) model had the highest predictive performance (AUC = 0.8). In this study, LR was found to be the second best‐performing ML model with a slight difference in accuracy from the Ada (AUC = 0.797).^10^

As LR is sometimes considered a traditional model and not an ML algorithm, a meta‐analysis concluded that ML was superior to the LR model in terms of mortality prediction after cardiac surgery, with a nonsignificant trend toward the better predictive ability of each ML algorithm. Nevertheless, the clinical importance of such an enhancement remains challenging.^35^ In contrast, another meta‐analysis reported that the discrimination of other alternative ML models for clinical prediction modeling was superior to the traditional LR in studies at high risk of bias, while this result was no longer in studies with low risk of bias.^36^

The frequency of hypertension among patients who require CABG is notable, as a recent study reported that hypertension was present in 54.6% of patients who underwent CABG.^37^ Moreover, hypertension is a significant risk factor for mortality and worsened prognosis after CABG.^9^, ^38^ Therefore, this study focused on the prediction of mortality after CABG in a hypertensive group of patients, that the LR model represented the best discriminative performance for predicting the 1‐year mortality. The simplicity of implementation and regularization, good efficiency from a training perspective, and not being affected by small data noise and multicollinearity constitute the advantage of LR.^39^ It has been reported that LR performs as well as ML models in predicting the risk of CVDs, chronic kidney disease (CKD), diabetes, and hypertension.^40^

Similar to our study, several studies aimed to investigate implementing ML models in certain groups of patients after cardiac surgery. For instance, Zhong et al.^41^ revealed that the XGB was associated with overall better predictive ability in terms of AUC compared to other models for forecasting the 30‐day mortality in critically ill patients after cardiac surgery. Consistently, another study compared five ML algorithms for estimating the long‐term mortality risk in the older adults (>65 years old) group who underwent CABG. Based on their results, the XGB and multivariate adaptive regression spline (MARS) models yielded the best predictive performance before and after variable selection, respectively.^42^ Altogether, there are controversies in selecting the best model for predicting clinical outcomes and mortality.

Feature selection is widely applied to removing irrelevant and unnecessary data, thereby could improve the accuracy and understanding of the ML models.^43^ RF algorithm has been applied in many studies^44^, ^45^, ^46^ and found to perform better in classification prediction modeling compared to other methods in ML techniques.^47^, ^48^ Since the use of too many features can lead to a decrease in the model's performance, reducing the number of variables and taking the correlation of features into consideration are among the advantages of the RF model.^45^ Likewise, in this study, we used the RF feature selector technique to determine the top features. Based on our results, the ventilation time after the surgery was recorded as the most influential variable for predicting mortality, followed by baseline EF.

Consistently, in another study, LR, RF, and XGB models selected the mechanical ventilation time as an important perioperative factor for predicting mortality after CABG.^42^ Also, the prolonged mechanical ventilation requirement after cardiac surgery has been reported as a predictive factor for in‐hospital and long‐term mortality, with patients who were intubated for more than 21 days having significantly worsened long‐term survival compared to other patients in 1 year (88.9 vs. 70.9%, *p* = .03).^49^ Fernandez‐Zamora et al.^50^ also reported that prolonged mechanical ventilation (>24 h) postcardiac surgery was observed in 10%–20% of patients, and they represented most of the postoperative mortality.^50^ In a meta‐analysis, He et al. reported that prolonged mechanical ventilation time (>48 h) could be associated with a higher risk of ventilator‐associated pneumonia (VAP), averaging 35.2%. Also, VAP after cardiac surgery is related to poor prognosis with high mortality and long ICU stays.^51^ The notable reverse relationship between low EF and risk of post‐CABG mortality has also been frequently reported by other investigations,^52^, ^53^, ^54^, ^55^ with a dose–response relationship between reducing EF and risk of death has been revealed.^53^ So far, lots of previous studies reported the pivotal role of age,^56^, ^57^ impaired glucose^58^, ^59^, ^60^ and lipid profile,^61^, ^62^ Hb levels,^63^, ^64^ serum creatinine,^65^, ^66^ and BMI^67^, ^68^ in estimating the early or late post‐CABG survival and mortality.

Similarly, in this study, TG, age, creatinine, Hb, LDL‐C, total cholesterol, FBS, HDL‐C, and BMI were detected in order as other important features by RF. The importance of these factors has been shown in several studies using various ML methods or otherwise. Like our study, a recent survey reported the 25 important predictors selected by the RF algorithm for mortality after cardiac surgery, which include chronic HF, mechanical ventilation, sodium, blood pressure, Hb, age, creatinine, renal failure, dyslipidemia, and glucose.^10^ In another study, XGB selected the serum creatinine, weight, age, and EF as the most important predictor for in‐hospital and 30‐day mortality of cardiac surgery.^69^ Besides, age, renal disease, chronic heart failure, and hyperlipidemia were selected as influential factors of long‐term survival in elderly patients with CABG by various ML algorithms.^42^

We applied each ML model for each of the subgroups of hypertensive patients, with the results implying that the LR model had the highest predictive ability for females, the age range of 50–59 and 80–89, overweight patients and diabetic cases, and smokers. This can elucidate the beneficiary effects of ML prediction models for each subgroup of patients and result in better utilization of such models in clinical settings. Although there are not many studies in this regard, the study predicting mortality in the elderly population undergoing CABG compared five ML models and found that XGB had the best predictive ability.^42^ These findings might suggest that it should be considered a targeted approach to training the ML models for mortality prediction in each subgroup of patients. However, more studies are needed to confirm these primary findings.

Our study proposed a prediction model for the hypertensive population undergoing CABG. As it has been reported that hypertensive patients can contribute up to 80% of patients scheduled for coronary revascularization surgery,^70^, ^71^ our findings can have clinical applications in these highly susceptible cases. There is a need for regional models designed for specific populations based on their local demographic features. There have been studies conducted on this topic in CABG patients in Iran^72^, ^73^; however, this is the first study focusing on hypertensive patients. Moreover, we designed these models with a combination of demographic, clinical, and laboratory characteristics which are widely available in clinical settings, while the use of a combination of variables has been shown to be beneficial in the prediction.^74^, ^75^

While our study was based on the databank, which provides a large population's various demographic, preoperative, intra‐operative, and postoperative information, there were several limitations. First, we used data from a single heart center in Iran. Although we tried to address this issue by performing a separate analysis on the most recent 30% of data to assess the model's validity over time, multicenter registry studies and assessment of the prediction models on other centers in the country are required to strengthen the generalizability of findings. Second, since the missing data of some relevant input features were high, we discarded them. Moreover, the potential effect of not including confounding variables should be considered. It should also be noted that there were no ECG data and follow‐up laboratory data available in this databank. Like usual real‐world clinical data sets, our data set is also composed of imbalanced data, which is a main methodological challenge in ML models. Several approaches have been suggested to resolve this issue, including oversampling the minority group, undersampling the majority group, and lowering the prediction threshold.^76^, ^77^ To overcome the imbalance of mortality data, we modified the threshold and applied the SMOTE oversampling method, which is more frequently used for predicting meager outcomes such as mortality than undersampling methods due to retaining valuable data.^15^, ^78^

## 5 CONCLUSION

Five different predictions using ML models for 1‐year mortality after CABG in hypertensive patients were developed. After applying RF for feature selection, the 11 most important features for the mortality prediction were detected. Among them, the mechanical ventilation time and baseline EF were by far the most influential determinants. All ML models, including LR, XGB, ANN, RF, and NB, demonstrated acceptable predictive performance, with LR providing the greatest AUC. ML algorithms may pave the innovative way for early and accurate prediction of post‐CABG mortality in high‐risk groups, especially hypertensive patients. It could offer individualized tools for clinical decision‐making and management. However, the current study's findings warrant further validation by more studies.

## AUTHOR CONTRIBUTIONS

**Amir Hossein Behnoush** and **Amirmohammad Khalaji**: Design, manuscript drafting, data analysis, and revision. **Malihe Rezaee**, **Shahram Momtahen**, **Soheil Mansourian**, **Jamshid Bagheri**, and **Farzad Masoudkabir**: Data gathering and manuscript drafting. **Kaveh Hosseini**: Supervision, design, manuscript drafting, and critical revision.

## CONFLICT OF INTEREST

The authors declare no conflict of interest.

## Supporting information

Supplementary information.

## Data Availability

The data set analyzed in this study, along with the codes used to develop and evaluate machine learning models, are available upon reasonable request from the corresponding author.
