# Supplementary material for: Machine learning‐based prediction of 1‐year mortality in hypertensive patients undergoing coronary revascularization surgery
Source: Clin Cardiol. 2023 Jan 1;46(3):269–78. doi: 10.1002/clc.23963 (PMC10018097; doi:10.1002/clc.23963)
Supplement: Supplementary file 1 — Supplementary information. [file CLC-46-269-s001.docx]

**Supplementary Table 1.** Baseline Characteristics of the hypertensive patients in the study cohort

| **Variable** | **Total Cohort (n = 8,943)** |
| --- | --- |
| Age (years) | 68.27 ± 9.29 |
| Body Mass Index (kg/m^2^) | 27.85 ± 4.33 |
| Male | 5,711 (63.86) |
| Diabetes | 4,189 (46.84) |
| Dyslipidemia | 5,805 (64.91) |
| Family History of Cardiovascular disease | 3,453 (38.61) |
| Smoking | 1,227 (13.72) |
| Prior MI | 2,758 (30.84) |
| Prior HF | 242 (2.71) |
| COPD | 327 (3.66) |
| Prior CABG | 54 (0.60) |
| Prior PCI | 744 (8.32) |
| PVD | 196 (2.19) |
| Prior CVA | 765 (8.55) |
| Opium | 1,217 (13.61) |
| Off Pump Surgery | 889 (9.94) |
| FBS (mg/dl) | 113.05 ± 40.67 |
| EF (%) | 46.79 ± 8.91 |
| Hb (g/dl) | 13.63 ± 1.74 |
| LDL-C (mg/dl) | 95.47 ± 35.99 |
| HDL-C (mg/dl) | 37.13 ± 9.74 |
| Cholesterol (mg/dl) | 155.11 ± 43.54 |
| TG (mg/dl) | 151.98 ± 77.49 |
| Creatinine (mg/dl) | 1.02 ± 0.67 |
| Total Ventilation Hours | 14.27 ± 34.37 |

Data are presented as mean ± S.D. or number (%); MI: Myocardial Infarction, HF: Heart failure, COPD: Chronic obstructive pulmonary disease, PCI: Primary cutaneous intervention, PVD: Peripheral vascular disease, CVA: Cerebrovascular accident, FBS: Fasting blood glucose, LDL-C: Low-density lipoprotein cholesterol, HDL-C: High-density lipoprotein cholesterol, TG: Triglyceride, BMI: Body mass index, EF: Ejection fraction, Hb: Hemoglobin

**
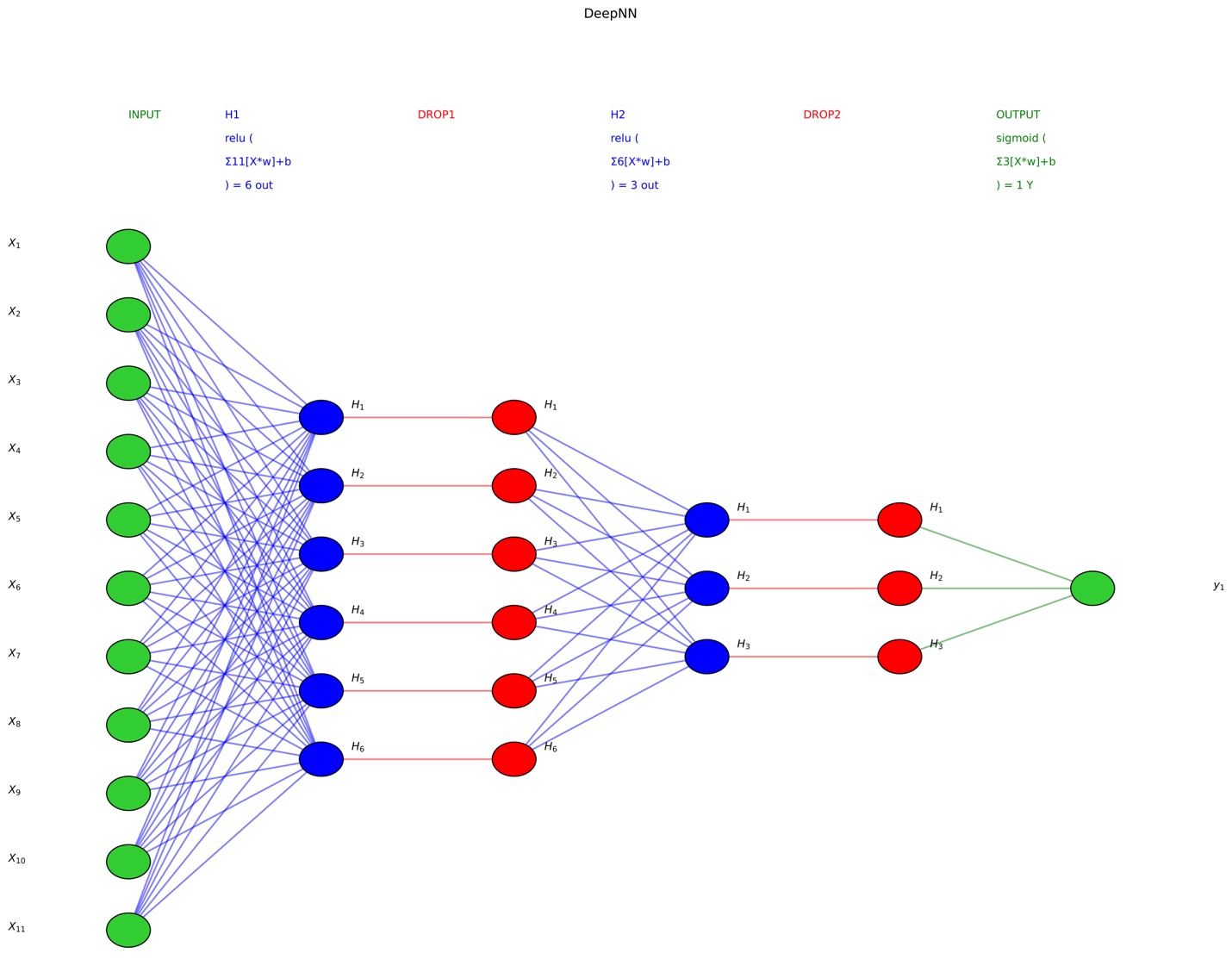
**

**Supplementary Figure 1.** Artificial Neural Network model diagram including the layers and nodes


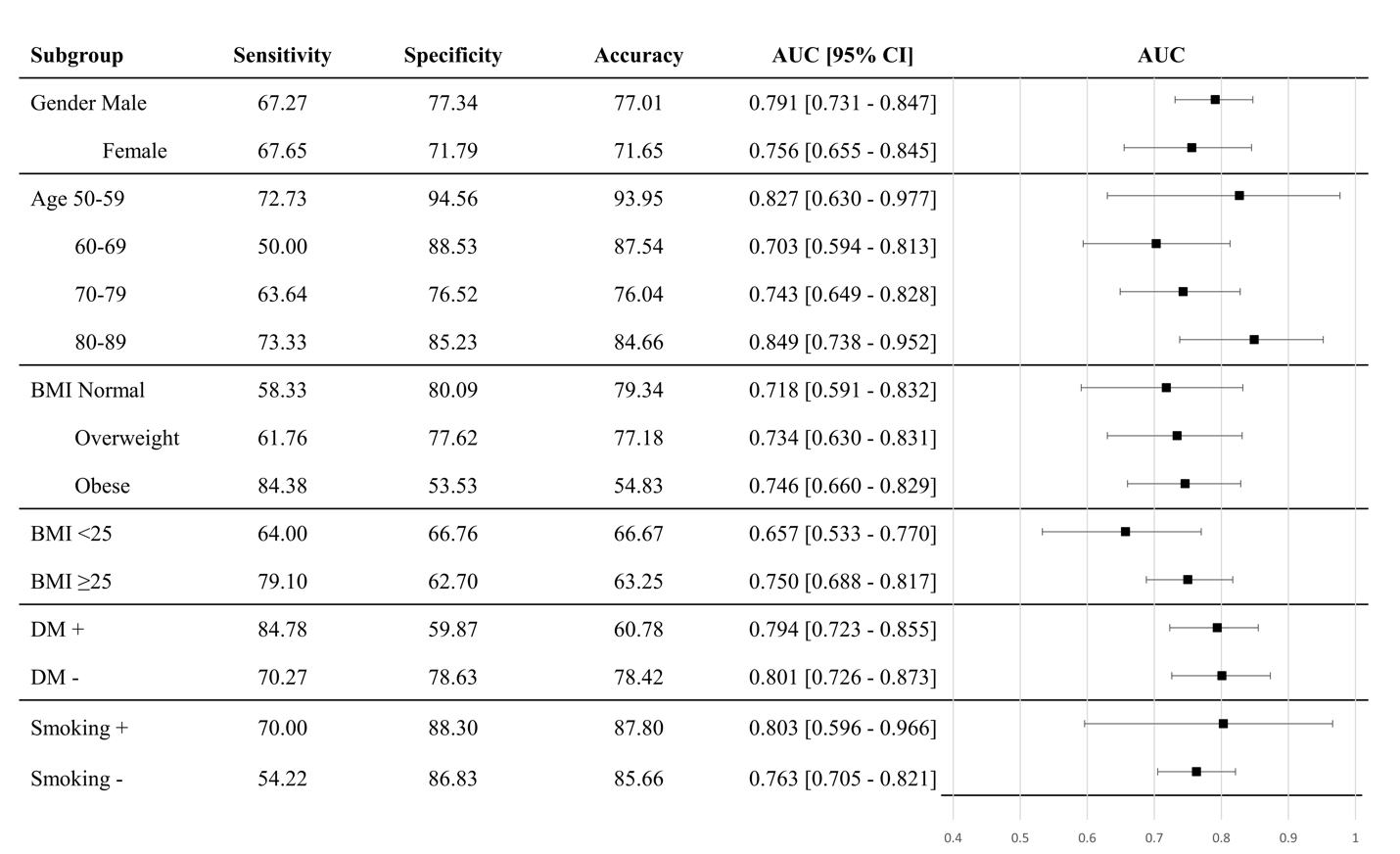


**Supplementary Figure 2.** Random Forest (RF) model evaluation for prediction of mortality in different subgroup of patients


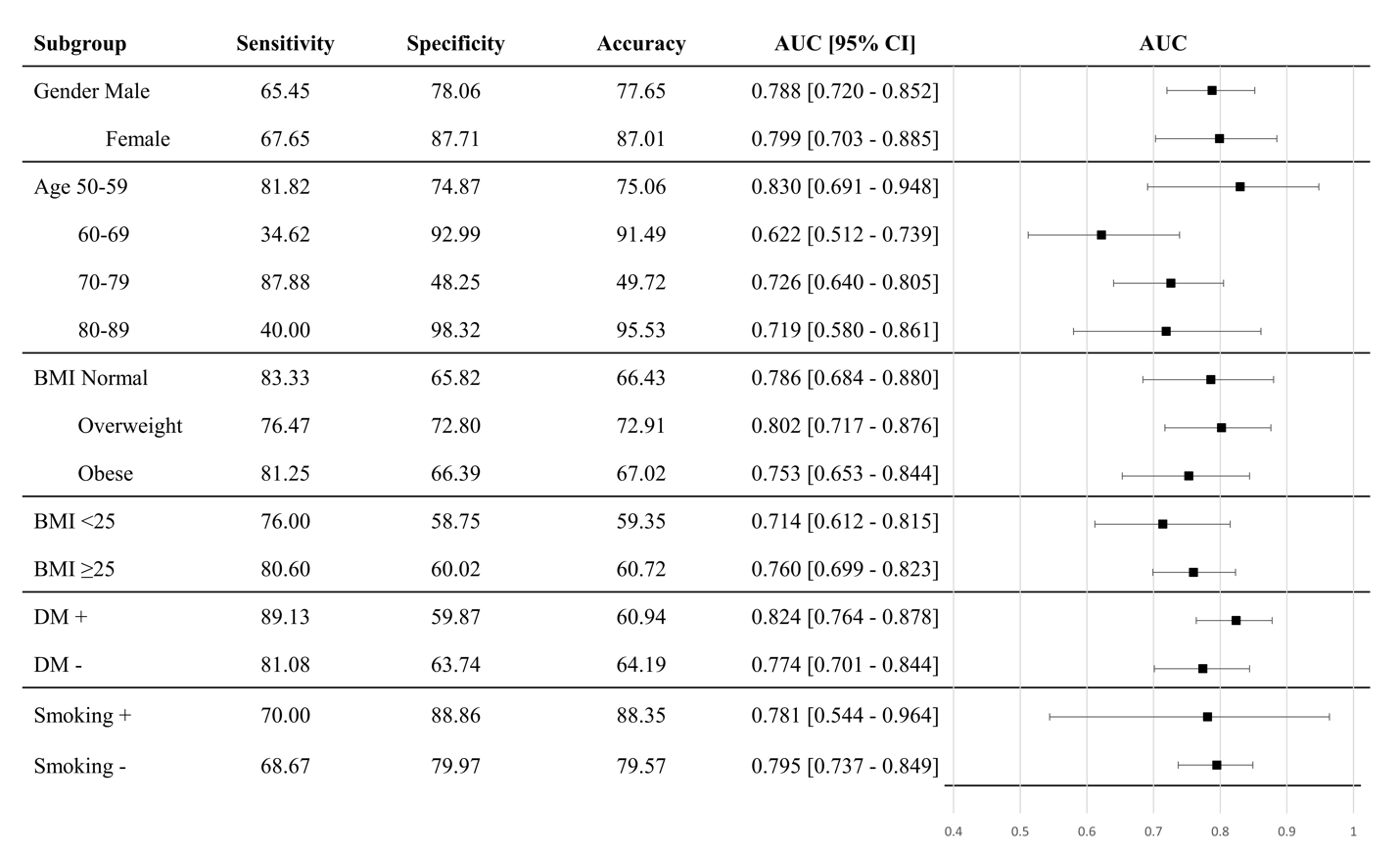


**Supplementary Figure 3.** Artificial Neural Network (ANN) model evaluation for prediction of mortality in different subgroup of patients


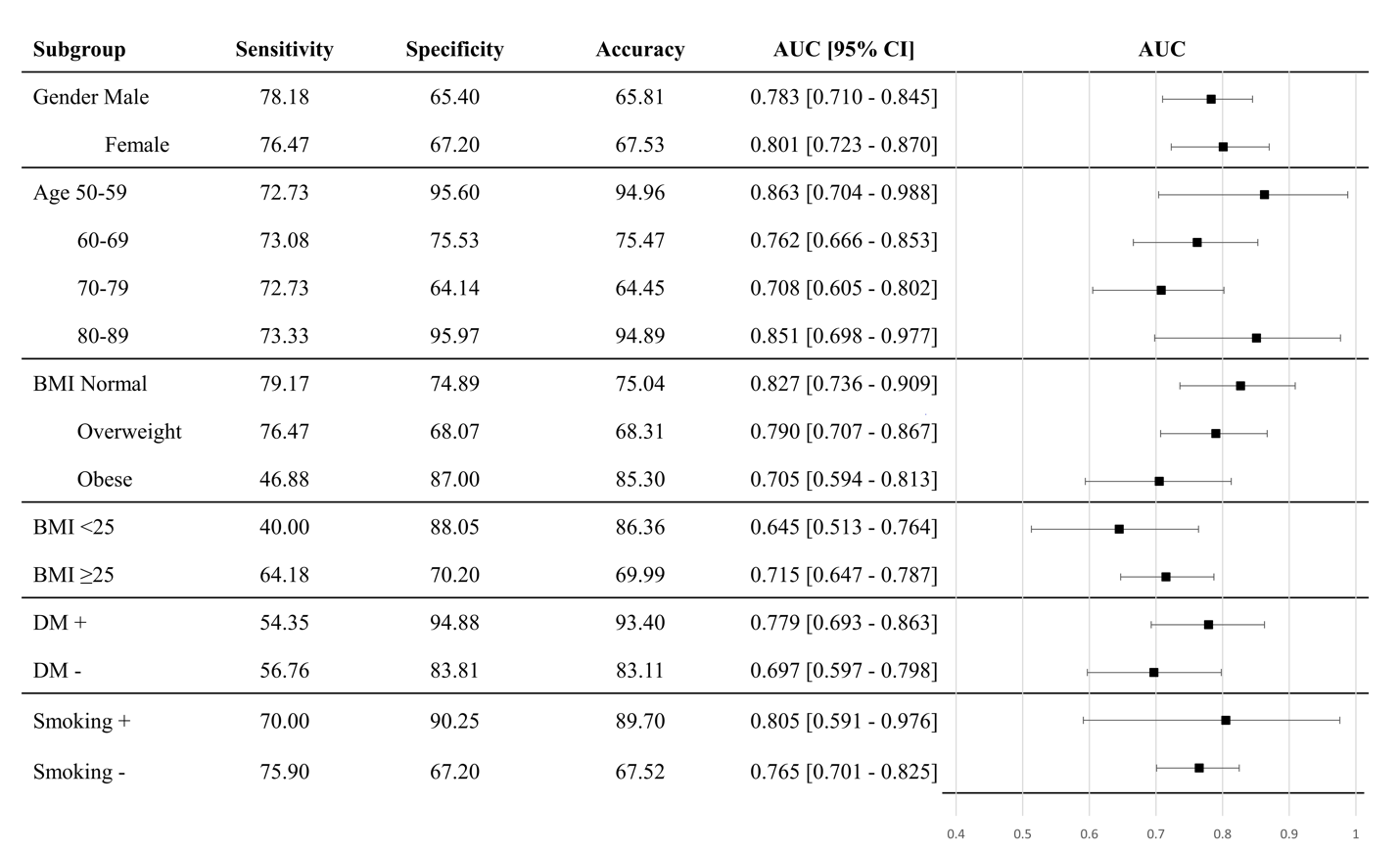


**Supplementary Figure 4.** Naïve Bayas (NB) model evaluation for prediction of mortality in different subgroup of patients


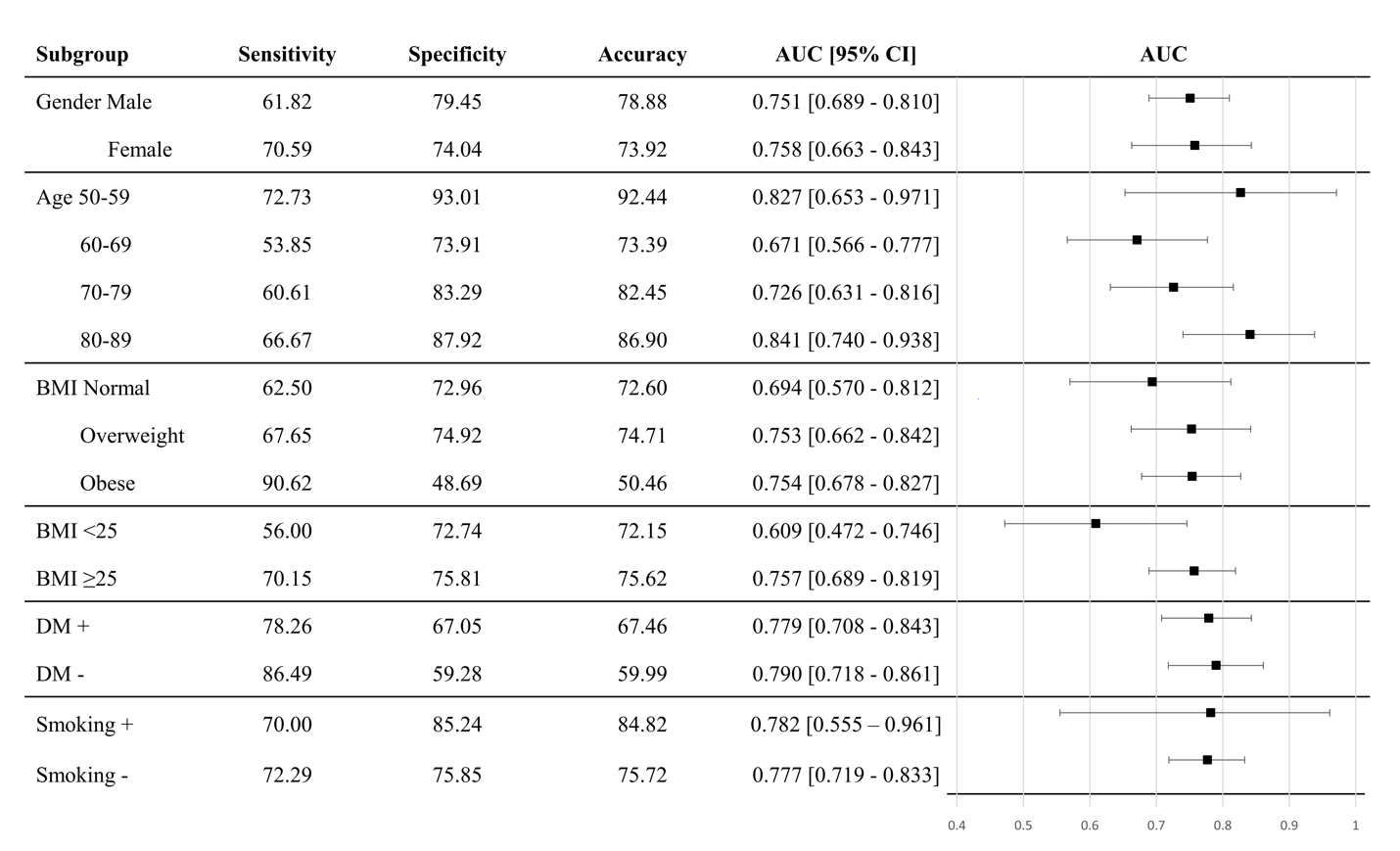


**Supplementary Figure 5.** Extreme Gradient Boosting (XGB) model evaluation for prediction of mortality in different subgroup of patients
